# Establishing a critical phosphorus dilution curve for potato in semi-arid regions based on a Bayesian analysis

**DOI:** 10.3389/fpls.2024.1458741

**Published:** 2024-09-17

**Authors:** Shuo Kong, Yonglin Qin, Xiaohua Shi, Jing Yu, Liguo Jia, Yang Chen, Mingshou Fan

**Affiliations:** ^1^ College of Agronomy, Inner Mongolia Agricultural University, Hohhot, China; ^2^ College of Grassland, Resources and Environment, Inner Mongolia Agricultural University, Hohhot, China

**Keywords:** potato growth, tuber yield, plant nutrition index, phosphorus status, Bayesian analysis

## Abstract

Phosphorus (P) fertilizer use efficiency in potato production is relatively low in semi-arid regions, wasting P resources and increasing environmental risks. Therefore, improving P use efficiency (PUE) is critical for sustainable potato production. The critical P dilution curve (CPDC) and P nutrition index (PNI) have proven to be robust diagnostic tools for assessing crop P status and aiding in improving P fertilizer management. Several potato CPDCs have been established, however, few studies have been conducted to establish a CPDC for potato (*Solanum tuberosum* L.) under ridge planting with drip irrigation, a planting pattern that has been increasingly adopted in semi-arid regions. In addition, the different CPDCs established using the conventional Jestus statistical model cannot discriminate the true variability across scenarios or have become linked to estimation errors. Therefore, the objectives of this study were to (1) establish a potato CPDC based on a Bayesian statistical method and (2) evaluate the relationship between potato yield and PNI. Three years of field experiments with five levels of P_2_O_5_ application (0, 80, 160, 240, 320 kg ha^−1^) were conducted in Inner Mongolia, China. No significant differences were found between CPDCs across the year × site for the assessed scenarios, and thus, a generic CPDC for potatoes in the region was derived as Pc = 0.616 DM^−0.296^, and it can be used to calculate the PNI. Further analysis showed that at each growth stage, the PNI exhibits a significant plateauing linear relationship with relative potato tuber yield. Thus, it provides a standard for diagnosing the P nutritional status in potatoes and lays a robust foundation for precise P recommendations in the region.

## 1 Introduction

Potato (*Solanum tuberosum* L.) requires a relatively large amount of phosphorus (P), with approximately 2 kg of P_2_O_5_ required to produce 1 t of tubers (Gao et al., 2011). However, potato has poor P absorption capacity because of its relatively shallow root system with low root density (Dechassa et al., 2003; Iwama, 2008). Moreover, P fertilizer applied to farmland is rapidly fixed by the soil (Holford, 1997). Thus, potato farmers often use P fertilizer in excess of what is required to maximize yields, and agronomists often recommend more than what is required (Rosen et al., 2014; Fan, 2019). This decreases P use efficiency (PUE) in the potato production system, increases potato production costs, as well as increases the accumulation of soil P, subsequently increases the risk of P enrichment in surface runoff water bodies, threatening the environment (Mekonnen and Hoekstra, 2018). In addition, recently studies demonstrated that constant accumulation of soil Olsen P beyond a critical value would lead to a significant increase in P leaching risk (Jalali and Jalali, 2017; Liu et al., 2023). Therefore, optimizing P fertilizer management in potato production has become an issue of particularly deep concern recently (Rosen et al., 2014; Fernandes et al., 2016; Soratto et al., 2020; Soratto et al., 2023; Huo et al., 2024).

Soil testing-based P fertilizer recommendations are commonly used worldwide. However, variability caused by soil type, climate, and cropping systems remain potential issues affecting the reliability of P fertilizer recommendations (Smethurst, 2000; Kleinman et al., 2001; Rayment, 2005; Cantarella et al., 2006). In the 1990s, it was proposed that measuring crop P or Pi content may address these issues (Campbell, 1994; Bollons and Barraclough, 1997; van Raij, 1998). However, P fertilizer was usually applied once during seed tuber sowing in most potato production regions; thus, potato P status evaluation was thought to be of less importance than potato N status evaluation, in which potato N status evaluation was often conducted to determine N fertilizer application rates during potato growth. Recently, a number of studies showed that P fertigation through drip irrigation systems can improve PUE (Eissa et al., 2019; Yin et al., 2005; Cui et al., 2020; Xing et al., 2015). As precipitation in semi-arid regions, such as Inner Mongolia in China, is often insufficient for potato production, irrigation is necessary to obtain ideal tuber yields and qualities. In addition, drip irrigation has been increasingly used in potato production because of its higher water use efficiency and convenience for supplying crop with fertilizer through irrigation. Therefore, split P fertigation to potatoes through drip irrigation systems is strongly recommended for potato production. In such case, plant P diagnosis is imperative for improving P management during potato growth.

The essence of crop tissue nutrient diagnosis is determining the minimum nutrient concentration (critical concentration) required for maximum biomass production in a crop at each growth stage (Ulrich, 1952). A mathematical model (Nc = A_1_W^-A2^) termed the critical nitrogen (N) dilution curve (CNDC) was first proposed by Greenwood et al. (1990) and then statistically improved by Justes et al. (1994) to serve as a critical tool to evaluate crop N status, where Nc refers to the critical N concentration, A1 refers to the plant %N when the biomass W equals 1 Mg ha^−1^ and A2 refers to the ratio of the reduction in plant %N relative to the crop growth rate. Once the model was proposed, it gained wide recognition, and CNDCs for more than 30 crop species in about 40 countries or regions have been established. Inspired by CNDC model construction, critical P dilution curves (CPDCs) for various crop species were similarly established, including timothy grass (*Phleum pratense* L.) (Bélanger and Ziadi, 2008), wheat (*Triticum aestivum* L.) (Bélanger and Ziadi, 2008; Bélanger et al., 2015; Li et al., 2022), cotton (*Gossypium* spp.) (Pang et al., 2020), maize (*Zea mays* L.) (Cadot et al., 2018; Liu et al., 2021), and potato (*Solanum tuberosum* L.) (Zamuner et al., 2016; Gómez et al., 2019). However, to the extent of our knowledge, a potato CPDC has not been established in semi-arid environments such as Inner Mongolia in China, where potato production is steadily increasing in Inner Mongolia, as well as in other semi-arid regions.

As CPDC is sensitive to the method used to estimate their parameters (i.e., type of statistical test and Type I error level), the Justes statistical method, which divides data into a restricted data group (with increased P application rates, plant dry weight (DW) and plant P content increase significantly) and a non-restricted data group (with increased P application rates, the DW and plant P content no longer increase) to identify critical P points as the intersection of the two linear regressions, has led to an increase in doubting whether variations in the model parameters reflect the true variability across genotype×environment×management (G×E×M) scenarios or are instead linked to errors due to sampling and parameter estimation (Makowski et al., 2020; Soratto et al., 2020; Ciampitti et al., 2021; Fernandes et al., 2021). For instance, Gómez et al. (2019) and Zamuner et al. (2016) established CPDCs for potato in Colombia (cv. Diacol Capiro: Pc= 0.523 W^−0.198^; cv. Pattusa Suprema: Pc= 0.536 W^−0.186^) and Argentina (cv. Innovator: Pc= 0.392 W^−0.304^), respectively using the Justes method. However, determining whether the difference among the A_1_ values of the three models, which varied from 0.329 to 0.536, originated from cultivar× region scenarios or parameter estimation is difficult. In addition, the Justes statistical analysis requires defining a classification rule to distinguish nutrient-limited versus non-nutrient-limited data so the fitted critical nutrient curve can be sensitive to these choices (Chen and Zhu, 2013). In such cases, there is increasing concern that false findings may be very frequent in research studies (Ioannidis, 2005); therefore, to limit the risk of false conclusions, a rigorous analysis of uncertainty is crucial.

Recently, a Bayesian statistical model was proposed by Makowski et al. (2020) for CNDC calibration and uncertainty analysis. The core of this method involves leveraging the Markov Chain Monte Carlo (MCMC) algorithm to construct a Markov chain random process for random sampling, continuously increasing the number of samples to estimate the parameters and confidence intervals. It considers all sources of uncertainty using the Markov algorithm and converts them into probability distributions (Gelman et al., 2014; Jégo et al., 2022). In contrast with the Justes method that is typically used to fit CNDCs, this method allows for direct curve fitting in a single step, avoiding the need to classify restricted and non-restricted data groups, and does not necessitate the preliminary identification of critical N concentrations. In addition, it circumvents the inability of parameter and curve-fitting confidence intervals to fully explain the uncertainty of selected critical concentrations (Makowski et al., 2020). This method is hypothesized to provide a more rigorous evaluation and has been successfully applied to some crop critical N concentrations in response to different G×E×M scenarios (Cheng et al., 2022; Jégo et al., 2022; Soratto et al., 2022; Li et al., 2024). To the extent of our knowledge, the concepts of CNDCs established based on Bayesian statistical analysis have not been applied to establish potato CPDCs. Therefore, one objective of this study was to establish a potato CPDC via the Bayesian statistical approach by collecting data from a 3-year field experiment with different P application rates in Inner Mongolia, China, and analyze the uncertainty in the resulting CPDC.

Once the CPDC is established, the minimal P concentration required to achieve maximum growth can be determined, then plant diagnostic methods of P deficiency can be developed on the definition of a critical P concentration. The P nutrition index (PNI), the ratio of measured P concentration to predicted critical P concentration which calculated from CPDC, can serve as an indicator to assess the crop P nutritional status during the crop growing season (Bélanger et al., 2015). PNI values less than 1 indicate that a crop would respond to P application, while PNI values greater than 1 indicate that a crop would not respond to P application. Therefore, the second objective of this study was to analyze the relationship between tuber yield and PNI and evaluate the possibility of diagnosing plant P status during potato growth using CPDC and PNI.

## 2 Materials and methods

### 2.1 Experiment site description

Field experiments were conducted in 2018–2020 in Inner Mongolia, China (41°30′N, 112°64′E). The frost-free period at the sites is ~100 d, the annual precipitation is ~300 mm, and the soil in the experimental fields is calcareous with a sandy texture. Drip irrigation was used in local potato production. The basic chemical properties of the soil 0–20 cm deep in the fields are presented in **Table 1**.

**Table 1:** Chemical properties of the soil 0–20 cm deep and rainfall during potato growth at the field experimental sites.

Year	pH	Organic matter (g kg^-1^)	Total nitrogen (g kg^-1^)	Olsen P (mg kg^-1^)	Exchangeable K(mg kg^-1^)	Rainfall (mm)
2018	7.8	27.3	1.46	9.5	124	230
2019	8.1	21.7	1.14	8.9	110	270
2020	8.3	24.4	1.48	18.4	145	280

### 2.2 Experiment design

In each experiment, five P fertilization rates were selected, and the treatment details are shown in **Table 2**. The P source was monoammonium phosphate. This experiment was performed using a randomized block design, with four replicates per treatment. Each plot covered 90 m^2^ and contained potato plants (cultivar Kexin-1) in rows 90 cm apart, with 20 cm between plants within a row. When sowing was performed, potassium sulfate was added to provide a K_2_O application rate of 285 kg ha^−1^. Urea was applied to provide a nitrogen application rate of 270 kg ha^−1^, with 25% of the urea broadcast at the time of sowing, and the remainder was split and applied through the drip irrigation system.

**Table 2:** General descriptions of the treatments (P rates), dates of sowing, harvest and sampling, nitrogen and potassium application rates.

Year	Sowing (d/m)	Harvest (d/m)	P rates (kg ha^-1^)	N rates (kg ha^-1^)	K rates (kg ha^-1^)	Sampling dates (DAE)
2018	3 May	1 Sep.	0,80,160,240,320	285	270	30, 45, 60, 70
2019	8 May	4 Sep.	0,80,160,240,320	285	270	30, 45, 60, 70
2020	3 May	4 Sep.	0,80,160,240,320	285	270	30, 45, 60, 70

DAE, days after emergence.

### 2.3 Plant sampling and measurements experiment design

For each treatment, four potato plants were sampled randomly 15, 30, 45, 60, and 75 days after emergence (DAE). Plants were divided into leaves, stems, petioles, roots, and tubers. After determining their fresh weights, the potato tissues were oven-dried to a constant weight for the subsequent analysis of P content. After grinding, each sample was digested in a mixture of H_2_SO_4_ and H_2_O_2_, and the total P concentration was determined spectrophotometrically using a Continuous Flow Analyzer System (SKALAR SAN++, the Netherlands).

### 2.4 Calculations

At the end of each experiment, the potatoes in 18 m^2^ of each plot were harvested, and the tuber yields were calculated as t ha^−1^.


Plant dry weigh (DW) = leaf DW + stem DW + root DW + tuber DW

Plant P accumulation = leaf P concentration× leaf DW + stem P concentration × stem DW + root P concentration × root DW + tuber P concentration × tuber DW

Plant P concentration = plant P accumulation / plant DW

$$\begin{array}{c} Relative\;yield\;=\;tuber\;yield\;following\;treatment\;/\;\\ maximum\;tuber\;yield\;for\;the\;experiment \end{array}$$

### 2.5 Model and statistical analysis

The potato CPDC was fitted using a Bayesian hierarchical model (Makowski et al., 2020). The Bayesian statistical model was implemented using R (R Core Team, 2024) and RStudio software (RStudio Team, 2023). During the fitting process, weakly informative prior (Model 1) was used to limit the influence of prior results, according to Makowski et al. (2020). Subsequently, the posterior distribution of the model parameters was estimated using the MCMC algorithm implemented in the R package rjags (Plummer, 2017). The MCMC algorithm for this model evaluated five chains, each with 40,000 iterations. After convergence was diagnosed using the Gelman–Rubin diagnostic, the first 40,000 iterations were discarded, and the MCMC algorithm was run for an additional 50,000 iterations to obtain the median and 95% confidence intervals (CIs) for the model parameters.

The SPSS 25.0 statistical software package was employed to assess the normality of these data and conduct tests for homogeneity of variance. ANOVA with Tukey’s test (P< 0.01) and regression analysis were performed. All figures were created using Origin 2021 software.

## 3 Results

### 3.1 The impact of phosphorus application rates on potato dry weight and phosphorus concentration

**Figure 1:**
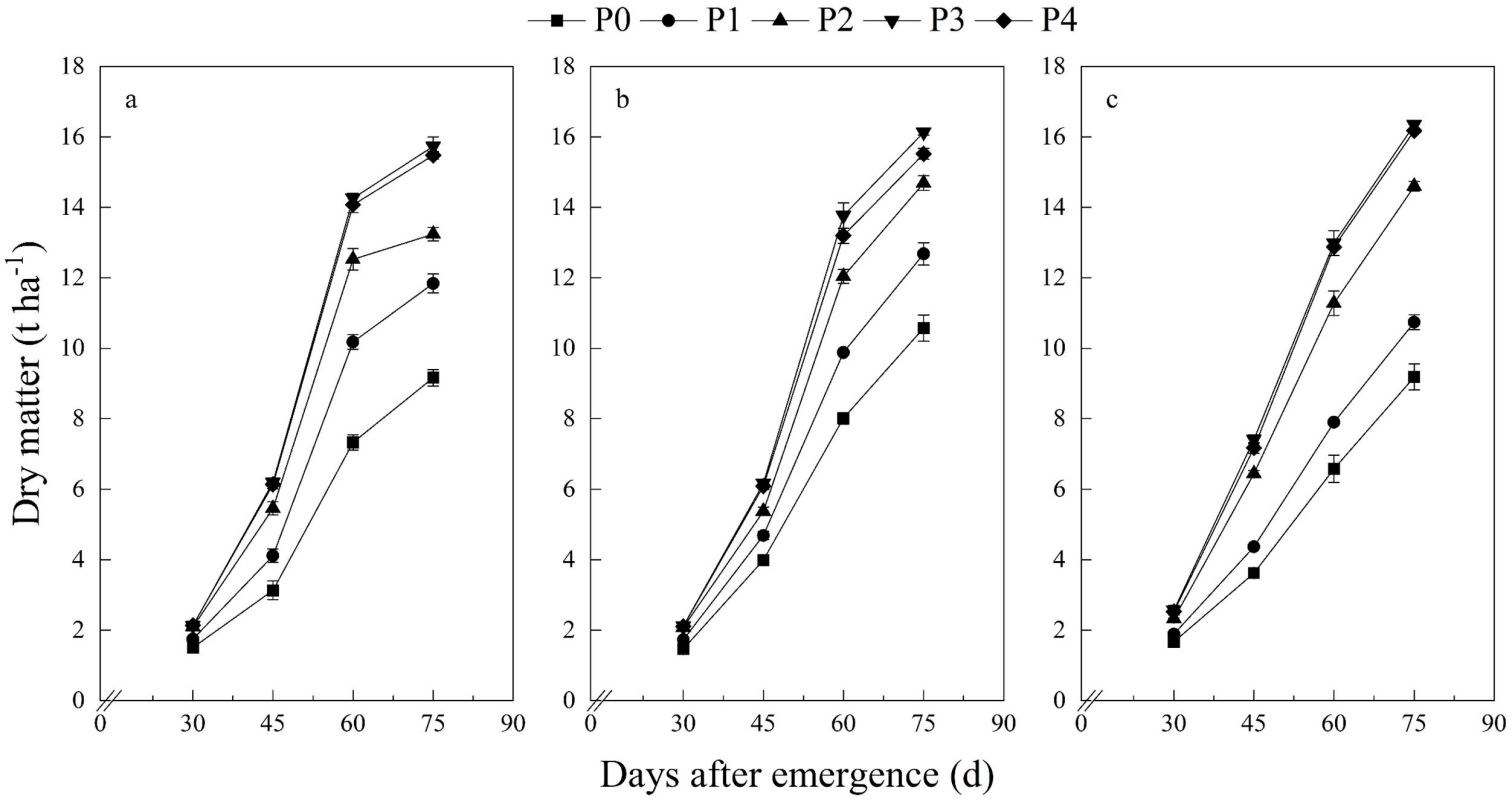
Potato plant dry matter at different growth stages. **(A)** 2018, **(B)** 2019, **(C)** 2020.

**Figure 2:**
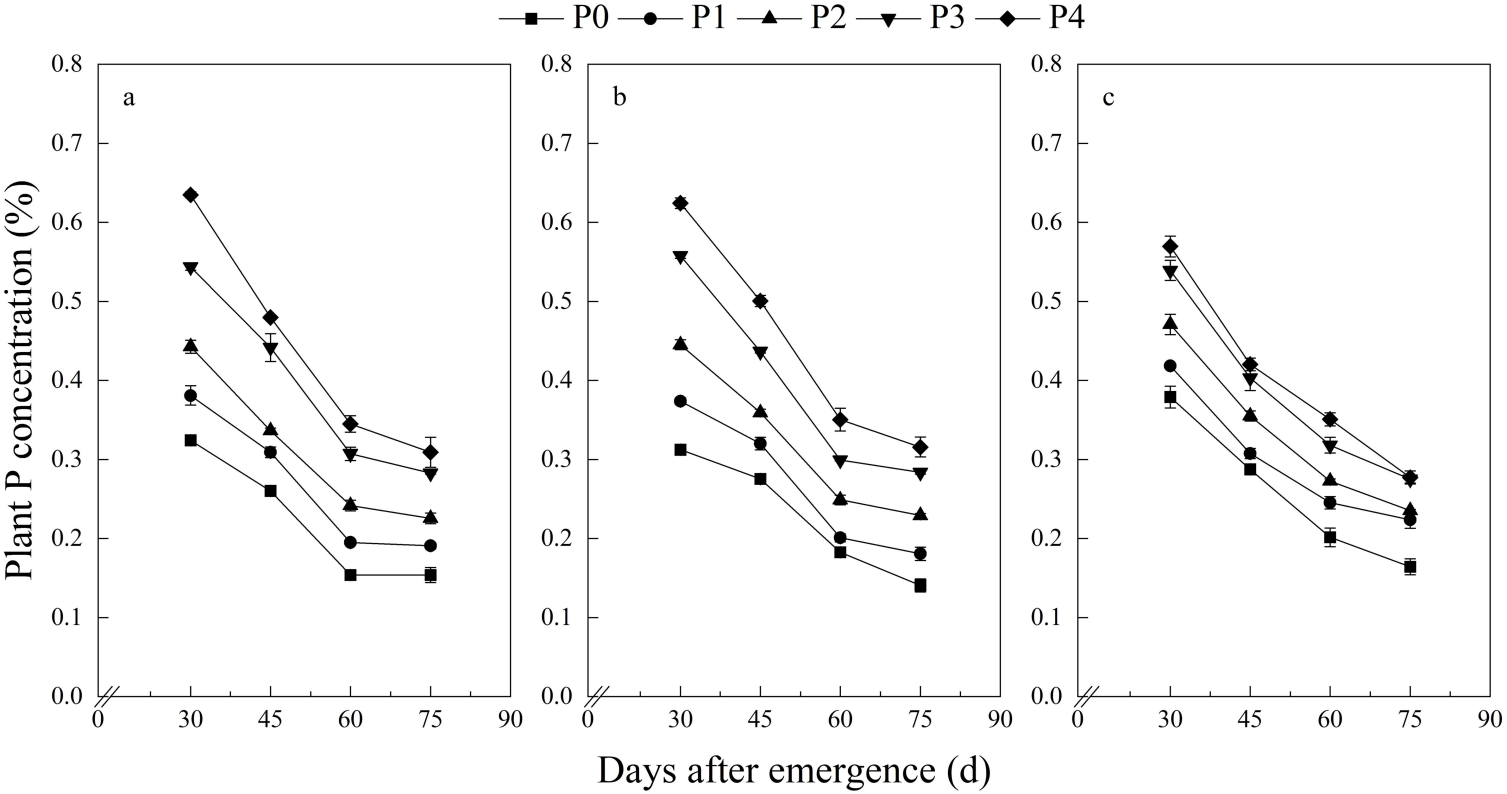
Potato plant P concentration at different growth stages. **(A)** 2018, **(B)** 2019, **(C)** 2019.

At each growth stage, the potato plants’ DWs increased significantly with an increase in P application rates (**Table 3**, **Figure 1**). The plant P concentration at each growth stage also increased significantly with an increase in P application rates (**Table 3**, **Figure 2**). In addition with potato growth, the DW of potato plants showed an increasing trend, with a consistent trend across three years (**Figure 1**). In contrast, the plant P concentration exhibited a gradually declining trend with the plant growth, consistent across all three years (**Figure 2**).

**Table 3:** ANOVA on potato dry matter weight and plant P concentration at different sampling times.

Sources of variance	Dry matter (t ha^-1^)				Plant P concentration (%)			
	30 DAE	45 DAE	60 DAE	75 DAE	30 DAE	45 DAE	60 DAE	75 DAE
Year	*	NS	NS	NS	NS	NS	NS	NS
Phosphorus rate	**	**	**	**	**	**	**	**

DAE, days after emergence; * and ** indicate significant at P_0.05_ and P_0.01_ respectively, and NS indicates insignificant at P_0.05_ level.

### 3.2 Establishment of the critical phosphorus dilution curve

**Figure 3:**
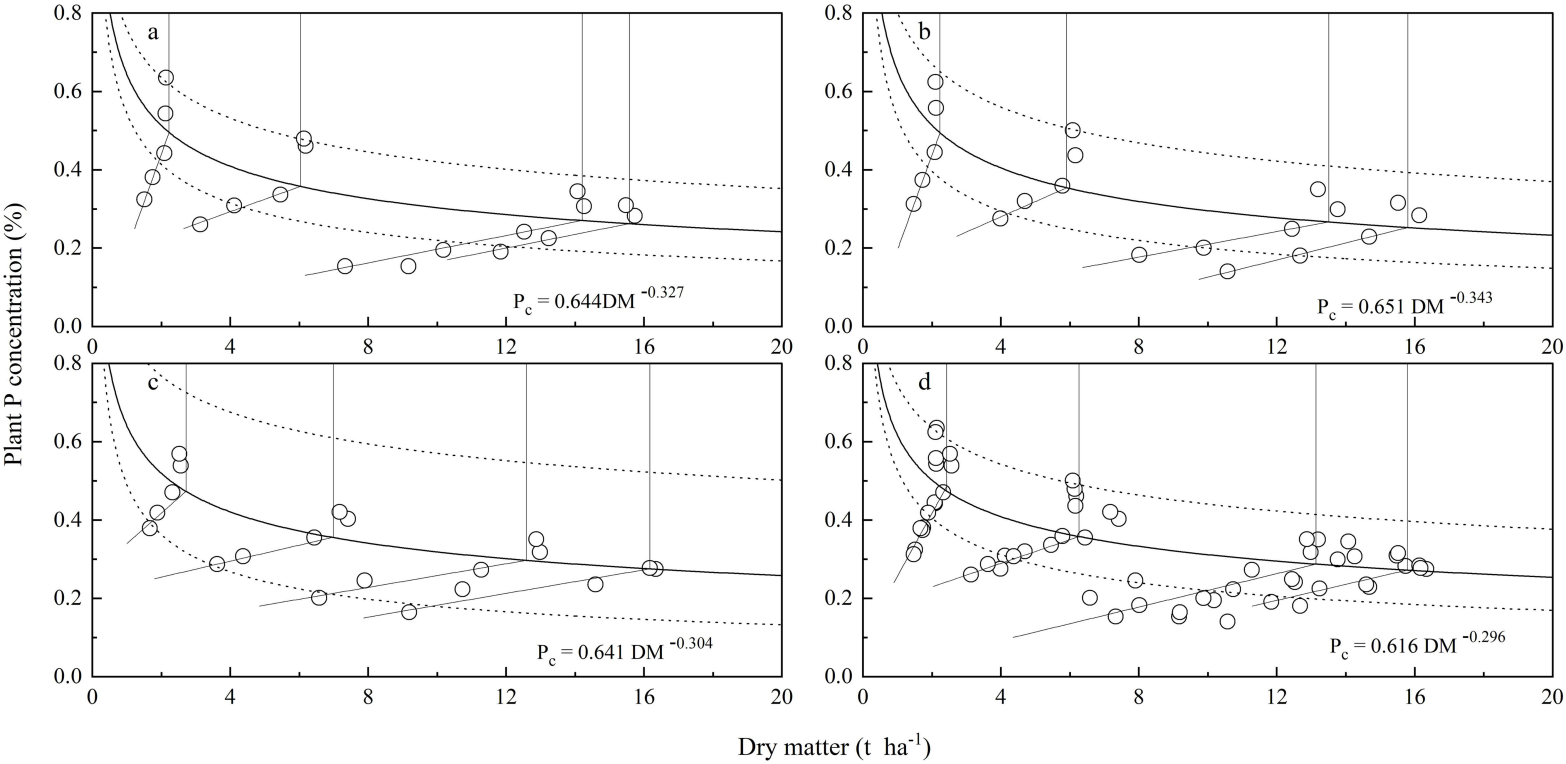
Critical phosphorus dilution curves (solid lines) and their 95% credibility intervals (dotted lines). **(A)** 2018, **(B)** 2019, **(C)** 2020, **(D)** for integrative analysis using pooled data of three years.

**Figure 4:**
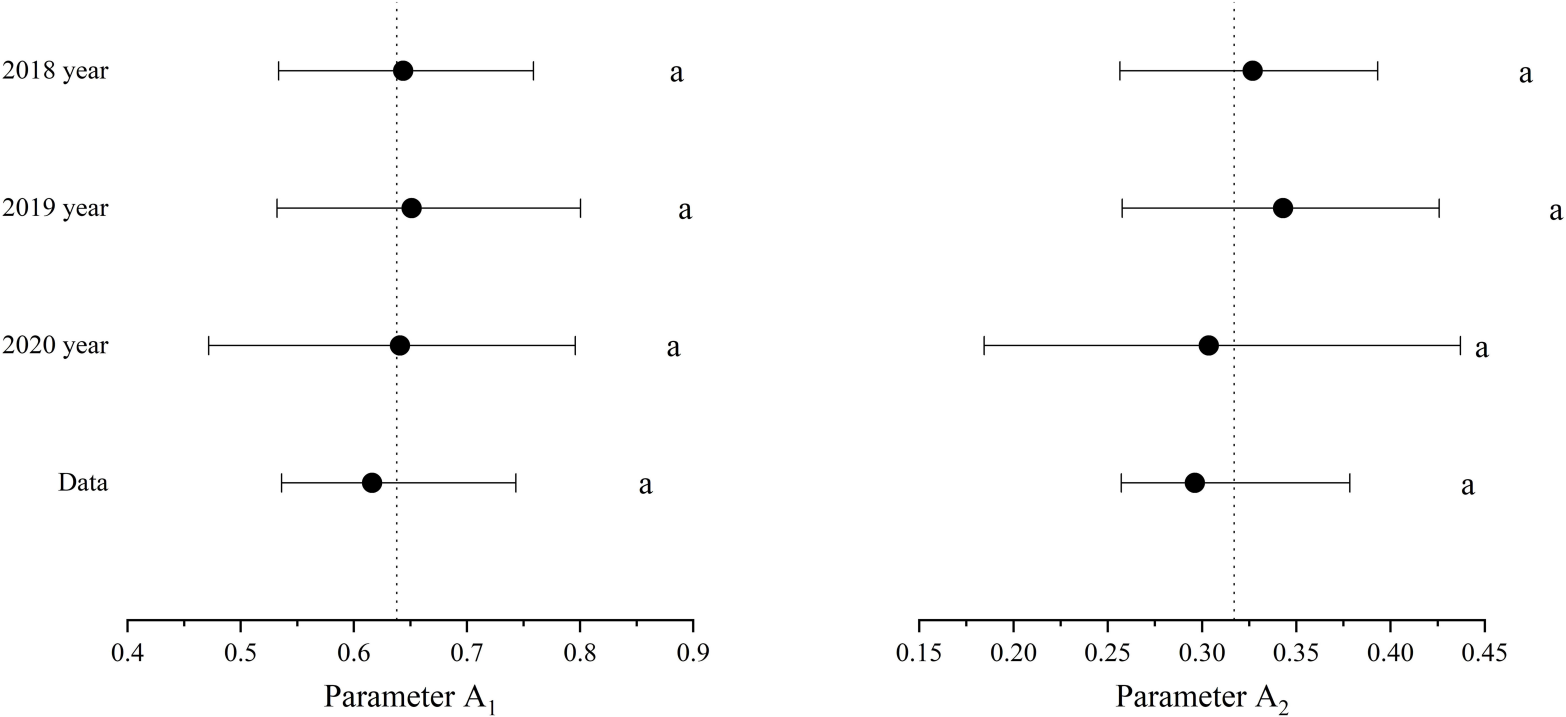
Estimated values (posterior medians) and 95% credibility intervals (CI) of parameters A1 and A2 through the Bayesian statistical model. The vertical dashed line represents the average of all critical posterior medians. Letters indicate significant relationships for the same parameter(95% CI). Data: all dates.

The CPDCs fitted using posterior values along with its 95% confidence interval are depicted in **Figure 3**, where the critical interval’s width describes the curve’s uncertainty. The uncertainty in the critical P concentration mainly arises from the DW, which gradually increases with an increase in plant dry matter weight (**Figure 3**). The median and 95% confidence intervals (CIs) of parameters A1 and A2 for the CPDCs are presented in **Figure 4**, where the interval plots depict the uncertainty in the parameters. The uncertainties for A1 of the CPDCs in 2018, 2019, and 2020 are 0.35, 0.41, and 0.51, respectively. For A2, the uncertainties in the corresponding years are 0.42, 0.49, and 0.83, respectively. The lowest relative uncertainties (0.33 for A1 and 0.41 for A2) were obtained when the CPDC was fitted to pooled data from the three study years.

### 3.3 The relationship between phosphorus nutrition index and relative yield

**Figure 5:**
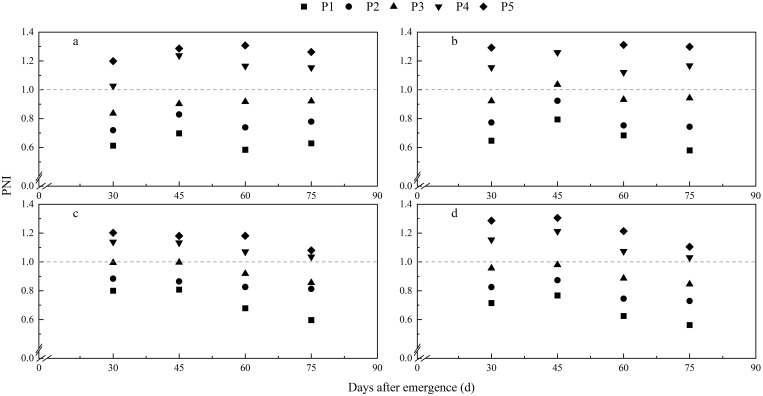
Dynamic changes of phosphorus nutrition index (PNI). Plotted using 2018-2020 data. **(A)** 2018, **(B)** 2019, **(C)** 2020, **(D)** for integrative analysis using pooled data of three years.

The potato PNI increased with an increase in P fertilizer application rate at each growth stage, and this was consistent across all experimental years (**Figure 5**). The PNI was less than 1 at each growth stage when the P application rates were less than 160 kg ha^−1^ (P3). When the P application rates exceeded 240 kg ha^−1^ (P4), the PNI at each growth stage was greater than 1 (**Figure 5**).

**Figure 6:**
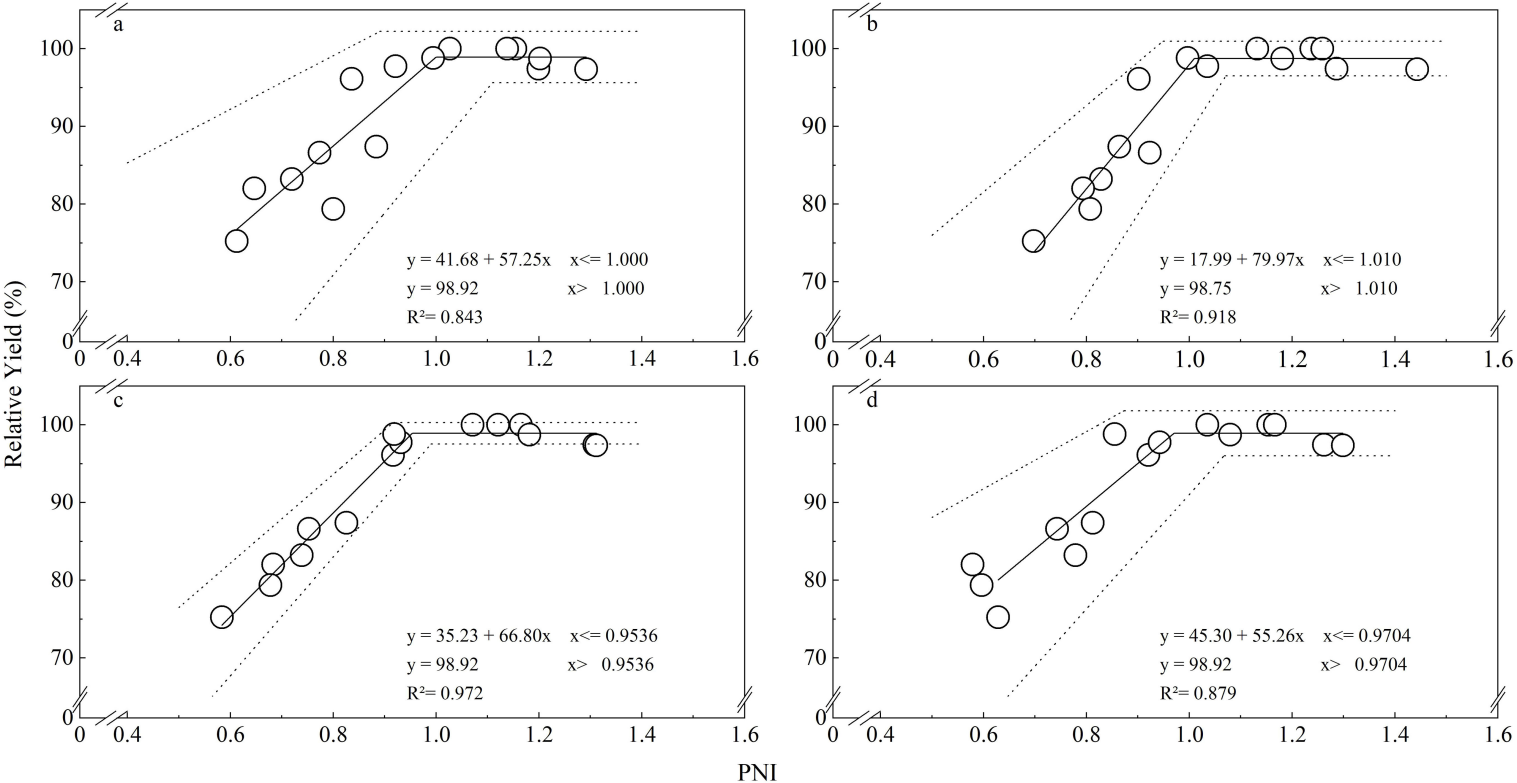
Relationship between PNI and relative yield and their 95% credibility intervals (dotted lines). **(A)** 30 days after emergence, **(B)** 45 days after emergence, **(C)** 60 days after emergence, **(D)** 75 days after emergence.

**Figure 6** illustrates the relationships between PNIs across the potato growing season and relative yield. The relative tuber yield exhibited a plateauing linear relationship with PNI at each growth stage (R²=0.843 ~ 0.972). The critical threshold values of PNI (the minimum PNI to reach the maximum relative tuber yield) at 30, 45, 60, and 75 DAE were 1.000 (95% CI = 0.8905, 1.1093), 1.010 (95% CI = 0.9487, 1.0712), 0.9536 (95% CI = 0.9169, 0.9902), and 0.9704 (95% CI = 0.8731, 1.0676), respectively.

## 4 Discussion

Similar to previous studies, this study showed that potato plant DW increased gradually with plant growth, while the P concentration was diluted with plant growth, with consistent results across three years (**Figures 1**, **2**). The variance in three CPDCs fitted using data sets from 2018, 2019, and 2020, respectively, was homogeneous (**Figures 3A–C**), suggesting that CPDC can be an integrative fitted and indicating that the effect of different year×site scenarios on CPDC was not significant within a region. Thus, a generic model Pc=0.616W^−0.296^ was derived based on data pooled from the three study years (**Figure 3D**), which lays the foundation for diagnosing potato plant P status using CPDC in the production region.

The average relative uncertainty is defined as the ratio between the width of the 95% CI and the medians of these two parameters (A1 and A2), respectively. The results of this study showed the relative uncertainties (0.19 for A1 and 0.35 for A2) obtained when the CPDC was fitted using the three-year data set (**Figure 3D**) were lower than any CPDC fitted using a single-year data set (**Figures 3A–C**). This not only confirms Fernández et al.’s (Fernandes et al., 2021) finding based on potato CPDC determination but also indicates that the Pc=0.616W^−0.296^ model is highly reliable.

As there is a close balance and synergy between N and P in crops (Lemaire et al., 2019), the N nutritional level affects the critical P concentration in crop (Bélanger and Ziadi, 2008; Bélanger et al., 2015; Nyiraneza et al., 2021). Previous local studies have identified potato critical N concentrations for each growth stage. For instance, at tuber bulking stage, the critical N concentration is 1.88~2.29 (Li et al., 2019; Zhang et al., 2020). In this study, the N concentration at tuber bulking stage was 1.80~2.27. Thus, the NNI at tuber bulking stage in this study was ~1. Similar situation was found for other growth stage. Additionally, local studies showed the maximum yield under N gradient conditions ranged from 49.82 to 57.96 t ha^−1^ (Li et al., 2019; Chen et al., 2024), while in this study, the maximum yield ranged from 53.36 to 54.88 t ha^−1^, suggesting no yield reduction due to nitrogen limitation. Therefore, the N supply in this study sufficiently met the requirement for potato growth, without affecting the establishment of the CPDC.

Values of PNI close to 1 indicate that crop growth is not limited by P supply, while values lower or higher than 1 indicate P deficiency and excess P consumption. The PNIs at each growth stage calculated in the present study using our Bayesian CPDC (**Figure 3D**) discriminated well between situations of P deficiency and excess consumption (**Figure 5**). This study found that the relative tuber yields were consistently related to the PNI at each potato growth stage (**Figure 6**), indicating that PNI can be used to guide potato P management development with the aim to achieve maximum tuber yield although tuber yield was not directly used to establish the CPDC.

## 5 Conclusions

Based on 3-year experiments in a semi-arid region, the CPDC for potatoes is established as Pc = 0.616 W^−0.296^ using a Bayesian hierarchical model. The PNIs at each growth stage calculated based on our Bayesian CPDC can discriminate well between situations of deficiency and excess P consumption, and the PNIs across the growing season all exhibit a significant plateauing linear relationship with relative potato tuber yield. Thus, this study provides a standard for diagnosing the P nutritional status of potatoes, laying a robust foundation for precise P recommendations in the region.

## Data Availability

The original contributions presented in the study are included in the article/**Supplementary Material**, further inquiries can be directed to the corresponding author/s.
