# Supplementary material for: Establishing a critical phosphorus dilution curve for potato in semi-arid regions based on a Bayesian analysis
Source: Front Plant Sci. 2024 Sep 17;15:1458741. doi: 10.3389/fpls.2024.1458741 (PMC11442389; doi:10.3389/fpls.2024.1458741)
Supplement: Supplementary file 1 [file Table1.docx]

**Appendix A. R Code**

library(readxl)

library(R2jags)

library(coda)

data <- read_excel(" ")

DM <- data$DM

P <- data$P

Date <- data$Date

unique_dates <- unique(Date)

Date_index <- as.integer(factor(Date, levels = unique_dates))

Q <- length(Date)

K <- length(unique_dates)

modelstring <- "

model {

# Weakly informative

Mu_DMmax ~ dnorm(16, 0.1)

Mu_S ~ dnorm(0, 0.1)

A1 ~ dunif(0.3, 1)

A2 ~ dunif(0, 0.5)

Prec_DMmax ~ dgamma(0.001, 0.001)

Prec_S ~ dgamma(0.001, 0.001)

tau_b ~ dgamma(0.001, 0.001)

tau_n ~ dgamma(0.001, 0.001)

for (i in 1:Q) {

DM[i] ~ dnorm(mu[i], tau_b)

P[i] ~ dnorm(Nc[Date_index[i]], tau_n)

mu[i] <- min(DMmax[Date_index[i]], DMmax[Date_index[i]] + S[Date_index[i]] * (P[i] - Pc[Date_index[i]]))

}

for (j in 1:K) {

Pc[j] <- A1 * DMmax[j]^(-A2)

DMmax[j] ~ dnorm(Mu_DMmax, Prec_DMmax) T(0,)

S[j] ~ dnorm(Mu_S, Prec_S) T(0,)

}

}

"

writeLines(modelstring, con = "model.txt")

model <- jags.model("model.txt", data = list(DM = DM, P = P, Date_index = Date_index, Q = Q, K = K),

n.chains = 5, n.adapt = 40000, inits = NULL)

gelman_rubin <- gelman.diag(posterior_samples)

gelman_rubin

posterior_samples <- coda.samples(model, c("Mu_DMmax", "Mu_S", "A1", "A2", "Prec_DMmax", "Prec_S", "tau_b", "tau_n", "S", "DMmax", "Pc"),

n.iter = 50000, thin = 10)

summary(posterior_samples)
